# Resveratrol—A Promising Therapeutic Agent with Problematic Properties

**DOI:** 10.3390/pharmaceutics17010134

**Published:** 2025-01-19

**Authors:** Lyubomira Radeva, Krassimira Yoncheva

**Affiliations:** Faculty of Pharmacy, Medical University of Sofia, 1000 Sofia, Bulgaria; l.radeva@pharmfac.mu-sofia.bg

**Keywords:** resveratrol, biological activities, nanoencapsulation, nanocrystals, prodrugs, structure analogues

## Abstract

Resveratrol is a natural polyphenol (stilbenoid), which can be found in grape skin, red wine, blueberries, peanuts and others. The biological properties of resveratrol, in particular antioxidant, anti-inflammatory, anticancer, estrogenic, vasorelaxant and cardioprotective activity, are the main reason for its importance in medicine and pharmacy. Despite all of its advantages, however, there are many problems related to this polyphenolic substance, such as low stability, water insolubility, poor bioavailability and fast metabolism. For this reason, scientists are currently searching for different approaches to dealing with these problematic properties and improving the therapeutic usage of resveratrol. This review summarizes the mechanisms of the biological effects of resveratrol, determined in vitro and in vivo, and the main limitations of the drug. The article emphasizes new approaches for the improvement of resveratrol delivery, in particular nanoencapsulation, formation of nanocrystals, prodrugs and structure analogues.

## 1. Introduction

Natural phenolic compounds have a variety of effects, namely antioxidant, anti-inflammatory, antibacterial and others. Resveratrol (3,5,4′-trihydroxy-*trans*-stilbene) is a natural phenol, more specifically a hydroxylated derivative of stilbene with a C_6_–C_2_–C_6_ structure. It is documented that resveratrol exists in two geometric isomer forms, and the trans-form has more pronounced antiproliferative effects as opposed to cis-resveratrol [1]. There are several sources of resveratrol—peanuts, grape skin, raspberries, blueberries, red wine, etc. It is produced by some plants in response to injury or fungal or bacterial attack. It was discovered that there is a non-specific response of the Vitaceae members to injury or infection where the biosynthesis of resveratrol occurs [2]. The compound may accumulate to between 50 and 400 μg/g fresh weight in leaves irradiated by ultraviolet light or being infected and is a paramount component (about 700 μg/g) of lignified stem tissue, but it could not be detected in healthy leaves.

**Figure 1:**
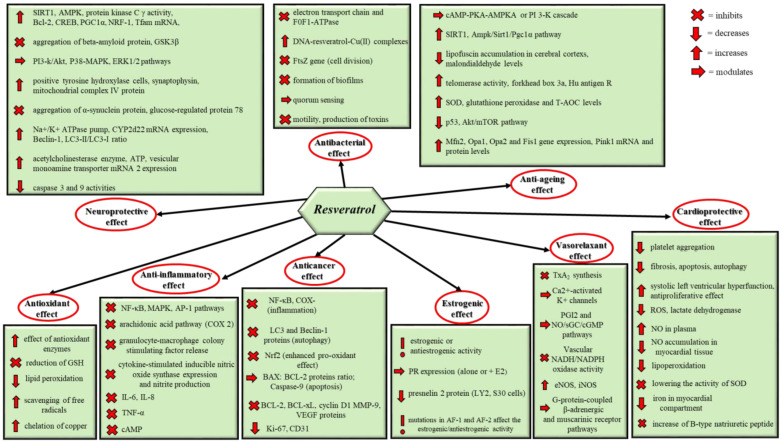
Examples of different mechanisms responsible for resveratrol’s positive effects.

Various biological activities of resveratrol are well-documented and are schematically presented in Figure 1. The most important ones are its anti-inflammatory effect, vasorelaxant activity, ability to modulate the lipid metabolism, anticancer and estrogenic activity, free-radical scavenging and chelation of copper, alteration of eicosanoid synthesis, inhibition of platelet aggregation and lipid peroxidation and antibacterial, antifungal and antivirus effects. Furthermore, one of the most significant characteristics of resveratrol is its cardioprotective activity [3,4,5,6]. These properties of resveratrol make it a promising therapeutic agent, although a possible toxicity and side effects were also summarized [7]. Despite its benefits, resveratrol is characterized with instability (isomerization and photodegradation), low water solubility, fast metabolism and poor bioavailability. For example, its oral bioavailability is less than 1% due to an extensive metabolism in the intestine and the liver. Also, it was reported that exposing trans-resveratrol to UV light can lead to isomerization to the cis-form [8]. Different approaches to dealing with the instability and low bioavailability of resveratrol were intensively investigated. Encapsulating resveratrol in nanosized drug delivery systems is one of the most researched strategies to enhance its stability and bioavailability. Numerous reviews have considered the advantages of nanoformulations, mainly for improvement of the anticancer potential of resveratrol [9,10]. It should be noted that there are important reviews on the effects and mechanism of action of resveratrol [11] as well as on clinical trials with its pure form [12]. However, recent studies applying other approaches that could overcome the limitations of the pure drug have to be summarized. For example, the use of resveratrol analogues may also improve its bioavailability [13].

Thus, the aim of the present review is to summarize the most significant effects and problematic properties of resveratrol and to update the approaches to overcoming its limitations. The review discusses the achievements and limitations, not only of nanoencapsulation but also of other strategies, including complexation, formation of nanocrystals, prodrugs and structure analogues.

## 2. Biological Effects of Resveratrol

### 2.1. Antioxidant Effect

**Figure 2:**
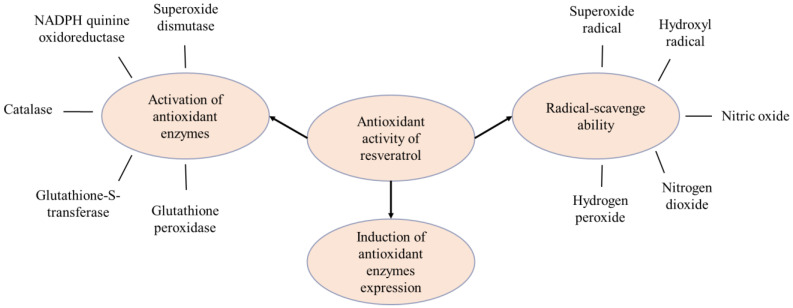
Mechanism of antioxidant activity of resveratrol.

Various studies have reported a strong antioxidant activity of resveratrol, which is related to its radical-scavenging ability (including superoxide and hydroxyl radical, hydrogen peroxide, nitric oxide and nitrogen dioxide), activation of antioxidant enzymes and induction of antioxidant enzyme expression (Figure 2) [14,15,16]. It was found that 3,4-dihydroxyl groups can enhance the antioxidant and anti-tumor activities of the polyphenol [17]. A study on the radical-scavenging ability of resveratrol and its analogues revealed that the para-hydroxyl group provided greater radical-scavenging activity of trans-resveratrol compared to its meta-hydroxyl groups [18]. In the same study, the activity of trans-resveratrol, vitamin E and vitamin C was also compared, and it was observed that resveratrol is the strongest scavenger. The trans-resveratrol also seemed to reduce lipid peroxidation by the scavenging of free radicals and the chelation of copper.

Trans-resveratrol, pterostilbene and quercetin were tested for their antioxidant activity in human erythrocytes in vitro [19]. Resveratrol showed a well-expressed inhibition of AAPH (2,2′-azobis(2-amidinopropane) hydrochloride)-induced hemolysis and prevention of the reduction of glutathione in AAPH-treated erythrocytes compared to the other two substances. Similarly, in vitro oxidative stress was induced in human erythrocytes by treating them with tetra-butyl hydroperoxide [20]. Resveratrol was discovered to have a protective effect, which was manifested by increasing the levels of intracellular reduced glutathione and sulfhydryl groups of the membrane.

### 2.2. Anti-Inflammatory Effect

The anti-inflammatory activity of resveratrol was explained by its influence on different signaling pathways that regulate inflammatory response, e.g., it inhibits the pathway of arachidonic acid, nuclear factor-κB (NF-κB), mitogen-activated protein kinase (MAPK) and activator protein-1 (AP-1) [21]. The mechanism of anti-inflammatory effect was studied in lung epithelial cells (A549) [22]. Resveratrol inhibited NF-κB, AP-1, cAMP (cyclic adenosine monophosphate) response element binding protein-dependent transcription (even more than dexamethasone), IL-8 (interleukin), granulocyte-macrophage colony-stimulating factor release, cytokine-stimulated inducible nitric oxide synthase expression and nitrite production. Furthermore, it is confirmed that, in the human mast cell line (HMC-1) treated with phorbol 12-myristate 13-acetate (PMA) plus calcium ionophore A23187 (Calcymycin), resveratrol inhibited the expression of IL-6, IL-8, TNF-α (tumor necrosis factor) and COX-2 (cyclooxygenase-2) due to its ability to diminish the Ca^2+^ and ERK ½ (extracellular signal-regulated kinases) levels in the cells [23].

### 2.3. Anticancer Effect

Regarding the anticancer activity of resveratrol, the pro-oxidant action and resulting apoptotic effect of the polyphenol are considered as responsible factors [14]. Possible mechanisms include the inhibition of the activity of phase-I-enzymes (CYPs), regulation the expression of nuclear factor erythroid 2–related factor 2 (Nrf2) and Nrf2 target genes, suppression of tumor cells’ growth, inhibition of angiogenesis by the regulation of VEGF (vascular endothelial growth factor) and metastasis and reduction of the proliferative capacity of cancer cells by altering their metabolism [24]. One of the functional groups of resveratrol, in particular the 4′-hydroxy group, is considered to be essential for its genotoxic activity (mutation of genes, aberration of chromosomes and DNA damage) [25]. The antitumor effect of resveratrol was examined in vitro and in vivo [26]. The synergetic effect of resveratrol in combination with doxorubicin was tested on MCF-7 and MDA-MB-231 cell lines (human breast cancer cells) and also on Ehrlich ascitic carcinoma (EAC) cell-bearing mice. It was discovered that resveratrol potentiates the cytotoxic effect of doxorubicin through inhibiting NF-kB and COX-2 (produced as a response to the inflammation), LC3, Beclin-1 proteins (which take part in autophagy) and Nrf2 (which participates in the regulation of redox stress). It also enhanced the apoptosis of the cells by altering the ratio BAX/BCL-2 (BAX is a protein that induces apoptosis, BCL-2 is a protein that blocks apoptosis). In vivo resveratrol inhibited the volume of the increased life span tumor. It was observed that the polyphenol could enhance the antitumor activity of gemcitabine in vitro on PaCa (pancreatic cancer) cells and in vivo on PaCa xenografted nude mice [27]. It inhibited proliferation, potentiated the apoptotic effects of gemcitabine and inhibited the activation of NF-κB and the expression of BCL-2, BCL-xL, COX-2, cyclin D1 MMP-9 and VEGF proteins. Furthermore, the combination of the drugs decreased the levels of the proliferation index marker Ki-67 and the density of micro vessel marker CD31.

### 2.4. Estrogenic Effect

The estrogenic activity of resveratrol is related to similarity in the structure of resveratrol and estradiol. The polyphenol may interact with estrogenic receptors (ERα and ERβ) and influence the action of the hormone. In particular, it exerts estrogen-agonist and antagonist effect in the absence of 17beta-estradiol or acts only as anti-estrogen in the presence of 17beta-estradiol [24]. Bowers et al. discovered that the polyphenol could act as an antagonist of ER-α but an agonist of ER-β [28]. The activity of resveratrol alone and in combination with 17β-estradiol (E2) was studied in cell lines, mammary glands of BALB/c mice placed in organ culture and after oral administration in nitrosourea-induced mammary tumors in rats [29]. Resveratrol possessed mixed estrogen agonist/antagonist activities in some mammary cancer cell lines, but in the presence of E2 it functioned as an antiestrogen. In the mouse mammary organ culture model, it induced progesterone receptor expression alone, but reduced this expression in combination with E2. In this model, resveratrol also suppressed the formation of estrogen-dependent preneoplastic ductal lesions, which were induced by 7,12-dimethylbenz(a)anthracene. Regarding oral administration in rats, the drug decreased the tumorigenesis provoked by N-methyl-N-nitrosourea. The effects of resveratrol and estradiol (E2) on the expression of exogenous reporter genes and an endogenous estrogen-regulated gene (TGFα) were compared in wild-type ERα transfected MDA-MB-231 cells or mutants with AF domains (transcriptional activation function domains), which were deleted or mutated [30]. The tests of exogenous reporter genes showed that resveratrol had superagonistic activity in these cells; however, the deletion of AF-1 and the mutation of AF-2 reduced this effect of resveratrol, and the deletion of AF-2 fully invalidated the response to both resveratrol and E2. The tests of TGFα expression also confirmed that resveratrol was an agonist in these cells. Furthermore, the deletion of AF-1 and AF-2 decreased the effect of E2 more than that of resveratrol, while the mutation of AF-2 limited the effects of both resveratrol and E2.

### 2.5. Vasorelaxant Effect

The vasorelaxant activity of resveratrol is related to various mechanisms, including inhibition of TxA_2_ synthesis, stimulatory effect on Ca^2+^-activated K^+^ channels, enhancing nitric oxide (NO) signaling and inhibition of vascular NADH/NADPH oxidase activity, increasing the expression of endothelial and inducible nitric oxide synthase (eNOS and iNOS) [31]. Resveratrol was tested on the resistance mesenteric and main uterine conductance arteries of female guinea-pigs on day 7 and day 15 of the oestrous cycle [32]. In the concentration range from 5 to 70 µmol/L, resveratrol showed a concentration-dependent vasorelaxant effect on both arteries, which were pre-treated with noradrenaline or potassium chloride (KCl). However, this effect was more manifested in the mesenteric vessels. The conclusions in the study were that the effect of resveratrol on resistance arteries is stronger than that on conductance arteries, the effects are not mediated via prostanoids (NO may play a role) and the vasorelaxant activity is not influenced by the oestrous cycle. Furthermore, an aortic ring assay model was used, where the rings were precontracted with phenylephrine in the presence of endothelium or with KCl in the absence of endothelium [33]. The study group used antagonists such as nonselective cyclooxygenase inhibitor (indomethacin), cyclic guanosine monophosphate lowering agent (methylene blue), selective soluble guanylate cyclase inhibitor (1H-[1,2,4]oxadiazolo[4,3-a]quinoxalin-1-one), nitric oxide synthase inhibitor (L-NAME), nonselective calcium activator potassium channel blocker (tetraethylammonium (TEA)), voltage-dependent K+ channel blocker (4-aminopyridine (4-AP)), inwardly rectifying K+ channel blocker (barium chloride), non-specific ATP-sensitive K+ channel blocker (glibenclamide) and β-adrenergic receptor blocker (propranolol). These antagonists significantly reduced the vasorelaxant effect of resveratrol in the presence of endothelium, especially indomethacin. Finally, the authors discovered that resveratrol blocked the calcium channels by reducing Ca^2+^ release from the sarcoplasmic reticulum, also affected potassium channels, PGI2 and NO/sGC/cGMP pathways, G-protein-coupled β-adrenergic and muscarinic receptor pathways.

### 2.6. Antibacterial Effect

The polyphenol is active against a wide range of bacteria, like Escherichia coli, Salmonella typhimurium, Vibrio cholera, Staphylococcus aureus, Bacillus cereus, Mycobacterium tuberculosis, Streptococcus pneumonia, Streptococcus pyogenes, Klebsiella pneumonia, Pseudomonas aeruginosa, Helicobacter pylori, Neisseria gonorrhoeae, Neisseria meningitides, etc. [6]. Some fungi are also reported as sensitive to resveratrol, particularly Candida albicans, Epidermophyton floccosum, Trichophyton tonsurans, Trichosporon beigelii, etc. [6]. The mechanisms of action include the reduction of a cell’s energy production by inhibiting the electron transport chain and F0F1-ATPase, damage of DNA by producing DNA-resveratrol-Cu(II) complexes, inhibition of cell division by the suppressing of the FtsZ gene, inhibition of formation of biofilms, modulation of quorum sensing, reduction of motility and production of toxins. For instance, resveratrol can enhance the activity of aminoglycosides against Gram-positive pathogens (Staphylococcus aureus, Staphylococcus epidermidis, Enterococcus faecium and E. faecalis), as well as to potentiate their action against biofilms produced by Pseudomonas aeruginosa [6,34]. Singh et al. observed that a conjunction of the 4′OH group with the methoxy group enhanced the antibacterial activity [35].

Some studies reported that resveratrol exerts eradication effect on bacterial biofilm by disturbing motility [36,37]. Moreover, applying a combination between resveratrol and the antibiotic colistin showed synergetic antibiofilm effect on colistin-resistant Pseudomonas aeruginosa, eliminated the multi-resistant bacteria in a murine infection model and enhanced the survival rate of infected *Galleria mellonella* [38]. The observed activity was due to disruption of the membrane of bacteria cells, which led to enhanced permeation and cell lysis. Thus, resveratrol alone or in combination with antibacterial agents may help in fighting the increasingly high resistance of bacteria to antibiotics.

### 2.7. Anti-Aging Effect

Resveratrol is also considered to possess anti-aging effects by the extension of lifespan. The putative mechanisms are related to its antioxidant effect, a decrease of albuminuria, inflammation and apoptosis, preservation of the mineral density of bones and the ability to enhance aortic elasticity and motor coordination. Furthermore, resveratrol inhibits aging-associated gene expression, modulates cAMP-PKA-AMPKA or PI 3-K cascade and activates members of the sirtuin family, especially SIRT1 [5,39,40]. The sirtuin family is a group of proteins (seven members in humans) that take part in aging-related pathological conditions by modulating cellular metabolism in stress conditions and modifying a variety of substrates [41]. Resveratrol reduced cognitive and oxidative damage (malondialdehyde levels), lipofuscin accumulation in the cerebral cortex, stimulated telomerase activity and the expressions of SIRT1 and downregulated p53 [42]. Another study group observed that resveratrol delayed ovarian aging [43]. Long-term treatment with the drug increased the number and quality of follicles and oocytes in aging mice, increased telomerase activity and SIRT1 expression and hindered the shortening of telomeres, and in low concentrations (0.1 µM) it enhanced cell proliferation in embryos. Thus, resveratrol may be a promising anti-aging agent that can prevent age-related diseases.

### 2.8. Neuroprotective Effect

Another remarkable activity of resveratrol is its neuroprotective effect. The polyphenol is active against many neurological disorders, such as stroke, dementia, sclerosis, Alzheimer’s, Parkinson’s diseases, etc. The neuroprotective activity is related to the antioxidant, vasorelaxant and anti-inflammatory effects of resveratrol. The ability of the drug to activate STIR1 and AMPK (playing an important role in the prevention of neuron apoptosis) and to inhibit the aggregation of the beta-amyloid protein also corroborates with the neuroprotection. Furthermore, resveratrol prevents the degeneration of dopaminergic neurons in substantia nigra by improving the number of positive tyrosine hydroxylase cells, inhibits aggregation of the α-synuclein protein and glucose-regulated protein 78 (a stress marker of endoplasmic reticulum in the brain), protects the Na^+^/K^+^ ATPase pump and the expression of CYP2d22 mRNA (it is considered to protect against Parkinson’s disease), increases acetylcholinesterase enzyme, ATP and the expression of the vesicular monoamine transporter mRNA 2 [3,44,45,46]. For instance, the action against oxygen glucose deprivation-induced mitochondrial dysfunction was investigated in vitro on SH-SY5Y cells. It was reported that resveratrol decreased caspase 3 and 9 activities, normalized cell viability by enhancing AMPK and p-AMPK’s activity and increased the expression of Bcl-2, CREB, mitochondrial genes PGC1α and NRF-1 [47]. It was discovered that in the amyloid-β protein precursor/presenilin 1 mouse model of Alzheimer’s disease, resveratrol hindered loss of memory (by the increase of the presynaptic protein synaptophysin), reduced the intensity and number of Aβ plaques and enhanced the levels of mitochondrial complex IV protein, the activity of SRT1 and AMPK pathways and the expression of IL1β and Tnf mRNA, which helped in plaque decrease [48]. Fonseca-Kelly et al. studied the possible effect of pure resveratrol and a pharmaceutical formulation of resveratrol with enhanced absorption (SRT501) on a multiple sclerosis model in C57/Bl6 mice [49]. In higher doses, both forms managed to delay the onset of the disease and neuronal loss, which led to better visual function. However, they could not decrease inflammation in the spinal cord and optic nerves and did not modulate the phenotype. In another study on a rat model of spinal cord injury, it was found that resveratrol improved locomotor activity and inflammation by the reduction of the pro-inflammatory cytokines TNF-α and IL-1β levels and the increase of the levels of the anti-inflammatory cytokine IL-10. Furthermore, the treatment with the polyphenol enhanced autophagy by increasing the level of the autophagy-associated proteins Beclin-1 and LC3-II and the expression of phosphorylated adenosine monophosphate-activated protein kinase (p-AMPK), which stimulates autophagy. It reduced the expression of the phosphorylated mammalian target of rapamycin (p-mTOR), which decreases autophagy. This leads to improved functional recovery and alleviation of inflammation, since it is considered that the induction of autophagy protects neurons from degradation while the inhibition contributes to neurodegeneration [50]. Thus, the neuroprotective effect of resveratrol is a vastly important activity that may advantage therapies, but it depends on various conditions and mechanisms of diseases.

### 2.9. Cardioprotective Effect

The cardioprotective effect is considered to be a result of the antiproliferative, antioxidant and anti-inflammatory action of the polyphenol (reduction of ROS and oxidation of LDL), modulation of various cell signaling pathways; regulation of vascular homeostasis, vasodilatation influence on the functioning of the endothelium and suppression of platelet aggregation [31,51]. To prove this function of resveratrol, its effect was tested on rats after ischemia and ischemia–reperfusion (I–R) [52]. The animals were pre-infused with or without resveratrol and after that rats’ left main coronary artery was occluded for 30 min or for 5 min, with a following reperfusion for 30 min. The aim of this research was to evaluate and to compare the intensity of the occurred arrhythmias and mortality. It was observed that resveratrol had much more intense protective effect against I–R-induced arrhythmias and mortality in contrast with only ischemia-induced ones. The authors considered that the incidence and duration of ventricular tachycardia and ventricular fibrillation were decreased because the pretreatment with resveratrol diminished lactate dehydrogenase levels but increased nitric oxide. The cardioprotective effect of resveratrol was also studied in lipopolysaccharide-treated rats [53]. The authors discussed that lipopolysaccharide from Gram-negative bacteria affects the heart and leads to septic shock and death. The cardiotoxic effect of this component is owing to a damage of DNA, proteins and membrane lipid due to the induced accumulation of reactive oxygen species. They concluded that the treatment with resveratrol decreased lipoperoxidation and myocardial nitric oxide raise, prevented lowering the activity of superoxide dismutase and stopped the transition of iron from plasma to the myocardial compartment. However, resveratrol had no impact on the increasing of peroxidase and decreasing of catalase. They suggested that iron-shuttling proteins may be included in the mechanism of the cardioprotective effect. Similarly, the mechanism of resveratrol’s cardioprotective effect in a murine isoproterenol-induced postinfarction heart failure model was explored [54]. It was found that resveratrol prevented hypertrophy, inhibited the increase of BNP (B-type natriuretic peptide) level in plasma, enhanced the systolic left ventricular hyperfunction, reduced interstitial fibrosis and had a positive effect on stress and prosurvival pathways, and it also normalized oxidative stress. Resveratrol was also found to be active in the treatment of malignant hypertension. The polyphenol was administered chronically in spontaneously and malignantly hypertensive rats [55]. It was found that it decreased the blood pressure via inhibiting lipid peroxidation, having a positive impact on oxidative status by the direct and enzyme-linked neutralization of ROS. In addition, it enhanced the release of NO, increased the superoxide dismutase, catalase and glutathione peroxidase enzymes, inhibited the myeloperoxidase and TGF-beta activity and suppressed the apoptotic process. Morphological changes in the heart tissues were also ameliorated [55].

The cardioprotective effect of the drug could be used with the aim of reducing the negative effects of cytostatic drugs on cardiomyocytes. Ehrlich Ascite carcinoma-bearing mice were treated with doxorubicin with or without resveratrol, and the cytotoxic and cardioprotective effects were evaluated [56]. Co-treatment with 15 mg/kg doxorubicin and 10 mg/kg resveratrol increased the survival rate and the mean survival time of the animals. Histopathological analysis of rat heart tissue after co-treatment showed no myocytolysis and fragmentation of the muscle fiber in contrast with single treatment with doxorubicin. Thus, resveratrol can enhance the cytotoxic effect of doxorubicin in tumor cells and may also protect the cardiac tissue from the cardiotoxicity of the anticancer drug. Furthermore, it was confirmed that the main ways by which resveratrol alleviates the cardiotoxicity of doxorubicin are by decreasing oxidative stress, apoptosis, cardiomyocytes autophagy and fibrosis induced by the antitumor drug [57]. Similarly, recent studies have confirmed these results and demonstrated an opportunity to improve the cardioprotective effect by simultaneous formulation of resveratrol and doxorubicin in composite nanogel particles [58] and polymeric micelles [59]. In conclusion, the positive effects of resveratrol are remarkable and could be further investigated and applied to improve the effectiveness of treatment strategies.

## 3. Limitations for Clinical Administration of Resveratrol

### 3.1. Low Aqueous Solubility

Despite all the advantages of resveratrol, there are some issues that hinder its application. One of these problems is its low aqueous solubility. The solubility of resveratrol in water is 0.05 mg/mL, which is considered as one of the reasons for its low bioavailability [60]. Higher solubility was found in alcohol (87.98 mg/mL) and PEG-400 (373.85 mg/mL) [60]. The solubility of resveratrol in different oils was also tested [61]. The authors concluded that the substance dissolves most intensively in coconut oil (179.75 ± 8.28 µg/g). Furthermore, by preparing oral and intravenous formulations of resveratrol with hydroxypropyl-β-cyclodextrin (HP-β-CD) and methylated-β-cyclodextrin (RM-β-CD), the solubility of resveratrol in water was improved, but neither the formulations nor the increasing doses coped with the low bioavailability [62]. Only RM-β-CD managed to increase the maximum plasma concentration when applied orally.

### 3.2. Low Stability

Another problem of resveratrol is its low stability under different conditions. It was discovered that resveratrol is more stable in acidic conditions (pH = 2–7) [60]. Particularly, more than 70% of the substance remained stable in acidic (pH = 5–6) and neutral conditions for more than 200 days, while increasing the pH value (8–10) led to only 2% remaining substance for about two months. Its basic hydrolysis is considered to be the reason for the degradation in alkaline medium. Another study also reported that in medium with pH > 6.8 resveratrol started to degrade, and the higher degradation level was at pH = 7.4 and 37 °C after 24 h [63]. They also observed that at pH values of 6.8 to 8 degradation followed first-order kinetics while at pH = 9 there were regions with first- and zero-order kinetics. Interestingly, at pH = 10 the profile was like an autocatalytic degradation process. The authors suggested that the mechanism depended on the degree of dissociation of the OH groups in the structure. In addition, there was a linear proportion between the degree of degradation and the temperature. Freezing the samples stopped the reaction due to decreased mobility of the molecules, while at 37 °C the process was rapid. The authors also studied the reaction of trans-resveratrol when exposed to UV light. After irradiation, resveratrol showed peaks at 306 nm (trans-resveratrol) and 287 nm (cis-resveratrol). The retention time of the cis-form was longer than that of the trans-form. López-Hernández et al. estimated the changes in the molecule of trans-resveratrol by irradiating it at 254 nm and 360 nm [8]. At 254 nm for 9 h and a distance between the sample and the lamp of 3.5 cm, the trans-form was converted to cis-form, but intriguing, the authors found an unknown compound, probably derived from the cis-form, whose peak was shown at the 30th minute. Exposure to UV light at 360 nm at a distance of 60 cm also led to the transformation of trans- to cis-resveratrol after 20 min of irradiation, and the unknown compound was also present. Most importantly, after 30 min under sunlight at noon there was a significant transformation to the cis-form, which depended on the intensity of solar light. This leads to the conclusion that the storage and transportation of trans-resveratrol could be difficult.

The photochemical and photocatalytic degradation of trans-resveratrol was also studied by using different TiO_2_ catalysts and irradiating it under different wavelengths (UV-Vis) [64]. The higher degradation rate was registered in the photocatalytic process, where six photoproducts were discovered. The products appeared mainly due to the attack of the hydroxyl radical (HO˙) to the substance’s molecule, which led to oxidation and fragmentation. The main degradation product was cis-resveratrol, and the transformation of the trans-form was proportional to the intensity of the irradiation. Corduneanu et al. evaluated the degradation and the transformation of resveratrol via voltammetry [65]. The authors reported that the oxidation of resveratrol is a pH-dependent process. In particular, a decrease of the oxidation potential with increasing pH of the solution was observed. The mechanism of oxidation includes two stages corresponding to irreversible oxidation of the phenol group and the resorcinol moiety.

### 3.3. Low Bioavailability

The low bioavailability is the main reason for the decreased activities of resveratrol in live organisms. The probable reason for this is the extensive metabolism of the substance [13]. The absorption, metabolism and bioavailability of ^4^C-resveratrol, applied p.o and i.v in six human volunteers, were tested [66]. It was observed that the absorption of a 25 mg oral dose was 70% or more and the half-life was 9.2 ± 0.6 h. Nevertheless, only less than 5 ng/mL of unconverted resveratrol was detected in plasma. Three metabolic pathways were discovered—sulfation of phenolic groups, glucuronidation of phenolic groups (both occurred in liver and intestine) and hydrogenation of the aliphatic double bond (probably induced by microflora in the intestines). Interestingly, most of the applied dose of resveratrol was identified in urine, and the extensive sulfate conjugation was considered to be the main reason for the low bioavailability. The bioavailability of trans-resveratrol in 25 healthy humans after moderate consumption of red wine was evaluated by applying three dietary approaches (fasting with standard meals, meals with high and low amount of lipids) [67]. Synthesized and purified trans-resveratrol 3- and 4′-glucuronides were used as standards, and the concentrations in the serum were determined via high-performance liquid chromatography (HPLC) with ultraviolet (UV) and mass spectrometry (MS) detection at different time intervals. It was discovered that only trace amounts of free trans-resveratrol were present in serum 30 min after wine ingestion, whereas the glucuronides were the main detected forms. The dietary regiment did not have any impact on the bioavailability of trans-resveratrol.

Similarly, the metabolic pathways of resveratrol in vitro (in human liver microsomes, human hepatocytes and rat hepatocytes) and in vivo (oral or intraperitoneal application to rats and mice) were studied [68]. No phase I metabolites were present in any samples and no metabolites were detected in the liver microsomes. The most common metabolites in rats’ urine, human and rat hepatocytes were trans-resveratrol-3-O-glucuronide and trans-resveratrol-3-sulfate, and also trace amounts of the cis-form were found. The problem with these metabolites is that they may have decreased or altered activity as compared to free resveratrol. For instance, the effects of trans-resveratrol and resveratrol-3-O- and 4′-O-glucuronides were compared in vitro on human peripheral blood mononuclear cells [69]. Free resveratrol had a cytotoxic effect in concentrations above 30 μM, but its metabolites did not have such an effect, even at higher concentrations (300 µM). The free resveratrol showed powerful synergistic anti-HIV activity, combined with didanosine at a concentration of 10 μM. However, the metabolites did not show such activity, even at a concentration of 300 µM. The authors concluded that this could not be fully accepted since in vivo a β-glucuronidase is present and it could convert 3-O- and 4′-O-glucuronides back into resveratrol. Furthermore, it is interesting that when comparing the estrogen agonist and antagonist effects of trans-resveratrol, trans-resveratrol-3-O-glucoside, trans-resveratrol-3-O-sulphate, trans-resveratrol-3-O-glucuronide and trans-resveratrol-4′-O-glucuronide, only resveratrol-3-O-sulphate showed strong oestrogen receptor α-preferential antagonistic activity [70]. This effect was also confirmed on human breast adenocarcinoma cell lines carrying a luciferase reporter gene under the control of oestrogen-responsive promoter.

The anticancer activity of resveratrol and its human sulfated conjugates (trans-resveratrol 3-O-sulfate, trans-resveratrol 4′-O-sulfate and trans-resveratrol 3-O-4′-O-disulfate) was studied in hormone-dependent breast cancer cell lines (MCF-7, ZR-75-1), the hormone-independent MDA-MB-231 cell line and one immortalized breast epithelial cell line (MCF-10A) [71]. It was discovered that all of the metabolites were less active than resveratrol against the cancer cell lines, and this was probably due to the inactivation of the phenolic groups of the drug. Thus, the in vitro studies indicated that any conjugation of the phenolic groups with sulfuric acid decreased the cytotoxic effect. Similarly, the sulfate conjugates of resveratrol showed a lower antiproliferative effect and remarkably low uptake in MCF7 cells [72]. It could be concluded that all the metabolites of resveratrol show altered activity in comparison with the free substance in vitro; however, the in vivo effects may differ significantly and should be further investigated.

All these problematic properties may hinder the pharmacological potential of resveratrol. For this reason, various new approaches intended to overcome its limitations are discussed in the following sections.

## 4. Approaches to Improving Resveratrol’s Application

**Figure 3:**
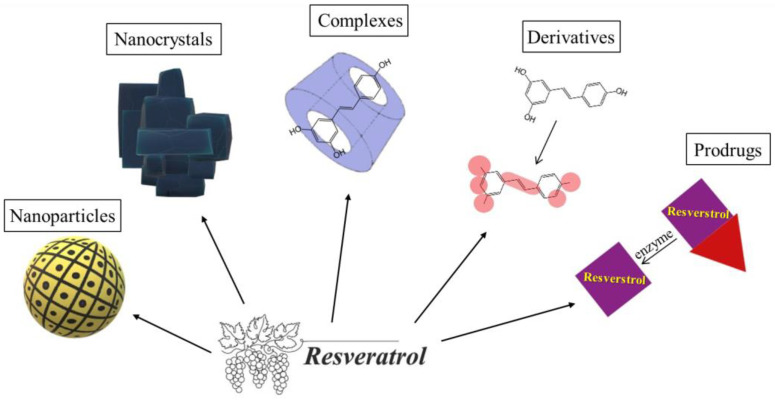
Approaches to resolving the problematic properties of resveratrol.

There are several promising approaches to dealing with the shortcomings of resveratrol, particularly encapsulating in nanoparticles, preparation of nanocrystals and complexes, usage of derivatives and analogues, and synthesizing prodrugs (Figure 3). Thus, the cited methods will be discussed with regard to their advantages, limitations and perspectives.

### 4.1. Encapsulation in Nanoparticles

**Figure 4:**
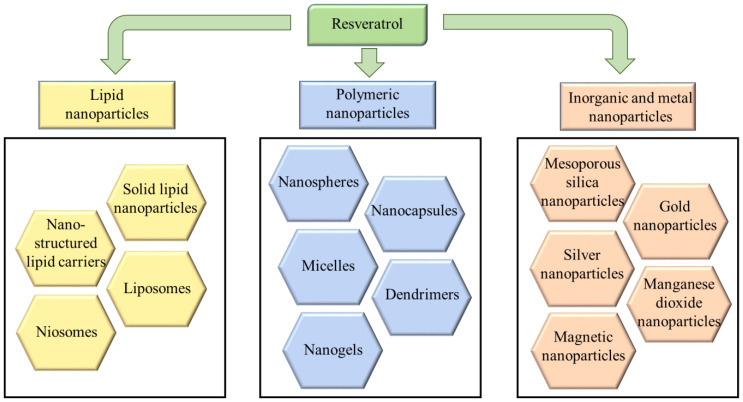
Types of nanoparticles investigated as drug delivery systems of resveratrol.

Encapsulating drugs in nanoparticles is a well-known approach to solve issues such as low solubility, stability, poor bioavailability, etc. Among various types of nanoparticles, the most investigated carriers of resveratrol are lipid, polymeric, mesoporous and metal nanoparticles (Figure 4).

#### 4.1.1. Lipid Nanoparticles

The lipid nanocarriers (solid lipid nanoparticles, nanosized lipid carriers, liposomes and niosomes) are widely used drug delivery systems since they are usually prepared from biocompatible and biodegradable lipids and could improve the stability, bioavailability, pharmacokinetic parameters, adsorption and permeability of the loaded drugs [73]. According to the literature, these nanoparticles are appropriate for the delivery of resveratrol (Table 1). For instance, resveratrol was loaded into solid lipid nanoparticles via the solvent emulsification evaporation method [74]. The resulting systems were characterized with approximately 127 nm mean size, negative zeta potential, haemocompatibility and sustained release. More importantly, the encapsulation ensured high biological half-life, lower clearance and improved biodistribution in prostate cancer cells compared to the non-encapsulated drug (7.56 times greater accumulation than that of the pure drug). Wang et al. reported the encapsulation of resveratrol in solid lipid nanoparticles obtained from D-α-Tocopheryl polyethylene glycol 1000 succinate, lecithin and stearic acid via the solvent injection method [75]. The nanoparticles (203 nm) provided increased cellular uptake of the drug, prolonged the cytotoxic effect and induced mitochondrial dysfunction in SKBR3/paclitaxel-resistant (SKBR3/PR) cells. The treatment of SKBR3/PR tumor-bearing mice with the encapsulated resveratrol demonstrated stronger suppression of tumor growth in vivo compared to the free drug. Solid lipid nanoparticles prepared from hydrogenated soybean lecithin, hydrogenated palm oil and sorbitol were loaded with resveratrol intended for oral delivery and improvement of insulin resistance [76]. The nanoparticles (248 nm, −16.5 mV) provided a stronger hypoglycaemic effect than the pure drug and restored serum oxidative stress status to the normal level in rats with type 2 diabetes. The authors hypothesized that the benefits of the encapsulated drug were due to the improved drug stability, intestinal absorption and increased circulation time of the encapsulated drug. Resveratrol-loaded nanostructured lipid nanoparticles (88 nm, 88% encapsulation efficiency) were prepared via the solvent injection method from stearic, oleic acid and Phospholipon^®^ 90 G [77]. The pharmacokinetic parameters, namely plasma concentration, half-life and clearance of resveratrol, were significantly improved after the encapsulation. Gadag et al. observed that the loading of resveratrol into nanostructured lipid nanoparticles, prepared from glyceryl monostearate and capryol 90 via melt-emulsification method, resulted in an enhanced anticancer effect, inhibition of the migration of MDA-MB-231 breast cancer cells and improved internalization. Furthermore, the systems showed an increase in the Cmax, Tmax and AUC_0-t_ of resveratrol when delivered via microneedles to the breast tissues in comparison with the pure drug administered orally [78].

The polyphenol was loaded into nanostructured lipid carriers and liposomes, achieving encapsulation efficiencies of approximately 86% and 96%, respectively [79]. The authors observed increased solubility, prolonged release and increased in vitro cutaneous permeability of resveratrol after the loading. Resveratrol-loaded liposomes were prepared from Tri-peptide lipid CDO (1,2-bis-myristyloxyamidopropyl ornithine) and sucrose laurate L1695 via the film hydration method [80]. The loaded liposomes (140 nm, 90% encapsulation efficiency) showed a pH-dependent release and improved anticancer effects in breast cancer MCF-7 cells and in BALB/c mice bearing MCF-7 breast cancer. Jagwani et al. prepared resveratrol-loaded cationic liposomes from soya lecithin, cholesterol and stearylamine by the film hydration method for treatment of hepatocellular carcinoma [81]. The liposomes (146 nm, 78% encapsulation efficiency) ensured an improved cellular uptake and anticancer activity in vitro in HepG2 cells compared to the free drug. In a rat model, the encapsulated drug had an increased internalization in cancerous rat liver tissues, confirmed by a 3.2- and 2.2-fold increase in AUC and Cmax, respectively. Multi-lamellar liposomes composed of phosphatidylcholine and Tween 80 were developed for the transdermal delivery of resveratrol [82]. The resulted liposomes (342 nm, 68% encapsulation efficiency) demonstrated an increase of the transdermal passing from 3% to 73% through excised human skin and to 10% through Strat-M™ artificial skin. Furthermore, the entrapment of resveratrol in the liposomes enhanced its stability by protecting it from isomerisation to its cis-form for up to 9 h. Resveratrol was also loaded into niosomes prepared from Tween 80 and Span 80 with/without dodecanol via the film hydration method [83]. The mean size was around 420/469 nm and the encapsulation efficiency was 57%/49%, respectively. The encapsulation managed to protect resveratrol from UV-C irradiation (15 min) and to decrease its isomerization to cis-resveratrol to 19% and 13%, respectively, in comparison with 42% for the ethanoic solution of the drug. The encapsulation of the polyphenol into Tween 80–Span 80 niosomes reduced the photoisomerization of trans- to cis-resveratrol even under UV irradiation for 1h [84].

Thus, the encapsulation of resveratrol in lipid nanoparticles results in increased solubility and stability, improved biodistribution, increased bioavailability and enhanced effects. However, some limitations of these types of nanoparticles have to be considered. For instance, the drawbacks of liposomes are related to low stability, oxidation of the phospholipids, short half-life, limited aqueous solubility and possible leakage of the encapsulated drugs [85]. Also, the cationic liposomes are known to possess cytolytic and cytotoxic activities [86]. Regarding solid lipid nanoparticles and nanostructured lipid carriers, various processes like polymorphic transitions, particle size growth, reticuloendothelial system clearance and accumulation in liver and spleen are possible [87]. Moreover, drug expulsion due to crystallization during storage and initial burst release could hinder the application of solid lipid nanoparticles [87].

**Table 1:** Types of lipid nanoparticle as carriers of resveratrol and achievements.

Type	Advantage	Reference
Solid lipid nanoparticles	Improved biodistribution	[74]
Solid lipid nanoparticles	Improved anticancer effects	[75]
Solid lipid nanoparticles	Alleviated insulin resistance	[76]
Solid lipid nanoparticles/ Nanostructured lipid carriers	Increased stability	[88]
Nanostructured lipid carriers	Improved pharmacokinetic properties	[77]
Nanostructured lipid carriers	Enhanced anticancer activity and improved pharmacokinetic properties	[78]
Nanostructured lipid carriers/ Liposomes	Increased solubility and cutaneous permeability	[79]
Liposomes	Improved anticancer effect and decreased organ toxicity	[80]
Liposomes	Improved anticancer effect and pharmacokinetic properties	[81]
Liposomes	Enhanced chemical stability and improved transdermal transport	[82]
Niosomes	Enhanced chemical stability	[83,84]

#### 4.1.2. Polymeric Nanoparticles

The wide variety of polymers that could be used for the preparation of polymeric nanoparticles provides a great opportunity for achieving drug delivery with defined characteristics and make these nanoparticles appropriate for different routes of administration. The encapsulation in such nanoparticles usually results in increased aqueous solubility and improved biodistribution, including the EPR effect. Furthermore, they could deliver the loaded drugs stimulated by different factors, such as pH, temperature and levels of oxidative stress. In addition, the nanoparticles could be modified in order to provide active targeted delivery. This is a prerequisite for increased bioavailability and effectiveness as well as for improved stability of the loaded drugs [89]. The main types of polymeric nanoparticles developed as carriers for resveratrol are nanospheres, nanocapsules, nanogels, micelles and dendrimers (Table 2). For instance, resveratrol has been loaded into β-lactoglobulin nanospheres, which possessed 165 nm average size, narrow size distribution (PDI 0.120) and negative zeta potential [90]. The encapsulation resulted in increased solubility and an improved anti-inflammatory effect in vivo. Cassano et al. loaded resveratrol into epigallocatechin gallate nanospheres, prepared via the microemulsion technique [91]. The systems were characterized with an average size of approximately 400 nm, PDI of 0.03, 100% encapsulation efficiency and enhanced anticancer activity against the MDA-MB 231 human breast cancer cell line. Three different types of starch nanocapsules (starch from Water chestnut, Horse chestnut and Lotus stem) have been obtained via the ultra-sonication method, and the encapsulation efficiency approximated 80% [92]. The anti-diabetic (inhibition of α-glucosidase) and anti-obesity (inhibition of pancreatic lipase and cholesterol esterase) activities of the encapsulated polyphenol were increased, especially when loaded into the Water chestnut starch nanoparticles. The authors concluded that the starch nanoparticles are able to protect the drug from the environmental conditions and to deliver it at its absorption site in the colon in its native form. Resveratrol-loaded composite nanocapsules from a γ-cyclodextrin-metal-organic framework and chitosan were obtained via the ionic gelation method [93]. The size of the formulation depended proportionally on the concentration of chitosan and was in the range of 194–322 nm, the zeta potential was positive and the PDI was below 0.3. The encapsulation efficiency also increased with the higher concentration of chitosan and reached approximately 91%. The loading of the polyphenol resulted in enhanced radical scavenge activity and increased photostability. Resveratrol was also loaded into zein-chitosan nanocapsules, achieving a 91% encapsulation efficiency [94]. The mean diameter depended on the concentration of resveratrol and was below 150 nm. The stability of resveratrol at 25 °C and 45 °C for 30 days of storage was significantly increased after the encapsulation.

Polymeric micelles also appear as advantageous drug delivery systems for resveratrol loading. Poly(methacrylic acid)-b-poly(ε-caprolactone)-b-poly(methacrylic acid) micelles were prepared via the solvent evaporation method for the oral delivery of resveratrol [95]. The drug loaded micelles were characterized with a 72% encapsulation efficiency, average size of 80 nm and negative zeta potential. The encapsulation resulted in increased solubility and enhanced the anti-inflammatory effect of resveratrol. In particular, the micellar resveratrol exerted a strong protective effect on epithelial HT-29 cells in a co-cultural model, representing the production of inflammatory cytokines, whereas the pure resveratrol did not protect the damaged HT-29 cells at all. Resveratrol was loaded into Pluronic F127—d-α-tocopheryl polyethylene glycol 1000 succinate nanomicelles via the film hydration method [96]. The average size of the optimal formulations was around 21 nm, and the encapsulation efficiency was 94%. Increased AUC_0-t_ and improved brain distribution were observed after the encapsulation of the polyphenol. Mixed micelles based on Pluronic F127 and Pluronic P123 (33 nm) were loaded with resveratrol with high encapsulation efficiency (79%) [97]. The encapsulation of resveratrol into the micelles increased its solubility significantly and provided a better protective spatial working memory effect compared to the pure drug in rats with scopolamine-induced memory impairment. Micelles prepared from the copolymer Soluplus were loaded with resveratrol via the film dispersion method, reaching almost 99% encapsulation efficiency and a diameter around 50 nm [98]. The in vitro passive permeation and cellular uptake, in vivo corneal permeation, anti-inflammatory and wound healing abilities were significantly improved upon the encapsulation in the micelles.

Sugary maize dendrimer-like glucan was loaded with resveratrol, achieving a 1.4% loading rate [99]. The encapsulation resulted in an approximately 9.1-fold increase of the solubility, enhanced antioxidant activity and increased cellular uptake. Resveratrol was also entrapped in poly(amidoamine) dendrimer, and the system improved its solubility by almost 2600-fold compared to the pure drug [100]. In addition, storage stability, topical permeability, pharmacokinetic parameters and bioavailability of the polyphenol were also improved.

Nanogels are attractive drug delivery systems due to their deformable structure, which is considered advantageous for cellular uptake. Resveratrol was loaded in nanogel particles based on citric acid and pentane-1,2,5-triol [101]. The nanogel was characterized with approximately 95% encapsulation efficiency and 220 nm mean diameter. The incorporation of the polyphenol into the nanogel matrix resulted in improved dissolution and enhanced protective effects against H_2_O_2_-induced oxidative stress in neuroblastoma SH-SY5Y and fibroblast L929 cells. The protective effect of the encapsulated drug against iron/ascorbic acid-induced lipid peroxidation on rat liver and brain microsomes was stronger than that of the pure drug [101]. Nanogel prepared from chitosan was loaded with resveratrol, achieving 59% encapsulation efficiency [102]. The nanoparticles possessed a mean diameter of 140 nm and positive zeta potential. The nanogel managed to protect the polyphenol from UV-irradiation-induced degradation for 90 min. Resveratrol was included in nanogel based on thiolated sodium alginate cross-linked with calcium lactate [103]. The authors reported 90% encapsulation efficiency, average size in the range of 200–300 nm and negative zeta potential. The nanogel increased the pH-, temperature-, redox- and UV-dependent stability of the drug. Furthermore, the in vivo antioxidant effects of the encapsulated resveratrol were also improved in Caenorhabditis elegans. Encapsulation of resveratrol in thermo-/pH-sensitive folic acid-poly(N-isopropylacrylamide-maltodextrin) nanogel (109 nm) also enhanced the in vitro (in MCF-7 cells) and in vivo (in Ehrlich ascites tumor-bearing mice) anticancer effect of the drug [104].

Taking into consideration the reported studies, the encapsulation of resveratrol in polymeric nanoparticles could be considered an appropriate approach to overcome its limitations, and specially to increase its stability. However, the polymeric nanoparticles possess some limitations. For example, because of the hydrophilic nature of nanogels the incorporation of hydrophobic drugs is challenging [105,106]. The drawbacks of dendrimers are related to molecular toxicity and immunogenicity, especially for cationic and higher-generation ones [107]. The successful loading of drugs into polymeric micelles is dependent on its affinity to the core-forming polymeric block [108,109].

**Table 2:** Various types of polymeric nanoparticles as carriers of resveratrol and their advantages.

Type	Advantages	Reference
Nanospheres	Improved biodistribution	[90]
Nanospheres	Improved anticancer effects	[91]
Nanocapsules	Alleviated insulin resistance	[92]
Nanocapsules	Improved pharmacokinetic properties	[93]
Nanocapsules	Enhanced chemical stability	[94]
Micelles	Increased solubility and enhanced anti-inflammatory effect	[95]
Micelles	Improved pharmacokinetic properties and brain distribution	[96]
Micelles	Increased stability Improved permeation Enhanced wound healing	[98]
Dendrimer	Increased solubility Enhanced antioxidant effects and cellular uptake	[99]
Dendrimer	Improved storage stability Enhanced topical permeability Improved bioavailability	[100]
Nanogel	Increased solubility Enhanced protection against oxidative stress	[101]
Nanogel	Increased photostability	[102]
Nanogel	Increased stability and antioxidant effect	[103]
Nanogel	Enhanced anticancer effect	[104]

#### 4.1.3. Inorganic and Metal Nanoparticles

Inorganic nanoparticles are distinguished with various advantages, particularly their higher stability comparing to the organic ones [110]. Therefore, the encapsulation of resveratrol into such nanocarriers could increase its solubility, stability and finally its effectiveness (Table 3). The most common inorganic nanoparticles developed as delivery systems of resveratrol are mesoporous silica. However, metal and magnetic nanoparticles are also perspective carriers. Resveratrol was loaded into mesoporous silica nanoparticles, achieving more than 93% encapsulation efficiency and a mean diameter of approximately 60 nm [111]. The aqueous solubility of the loaded resveratrol was increased, probably due to transition in amorphous form. Furthermore, there was a 2.36-fold decrease in the IC_50_ after 48 h treatment of melanoma MNT-1 cells with the encapsulated drug. Resveratrol was also encapsulated into lactoferrin-functionalized, hollow mesoporous manganese-doped silica nanoparticles via the adsorption method for treatment of ischemic stroke [112]. The systems possessed approximately 140 nm average size and 39% drug loading. The encapsulation resulted in improved antioxidant and anti-inflammatory effects in vitro in rat nerve cells, PC12, and mouse microglial cells, BV2. The in vivo studies in the middle cerebral artery occlusion model in rats showed an improvement of pharmacokinetic parameters (AUC_0-t_ and t_1/2_) and eventually enhanced bioavailability. Another study reports that resveratrol was loaded into mesoporous silica nanoparticles, which led to enhanced inhibition of the proliferation, invasion and migration and increased apoptosis of breast cancer MGF-7 cells [113]. The in vivo experiments in MGF-7 tumor-bearing mice revealed that the encapsulated drug enhanced the inhibition of breast cancer progression via inhibiting the NF-κB signaling pathway.

Gold nanoparticles were loaded with resveratrol and possessed an average size of approximately 39 nm and negative zeta potential [114]. The loaded formulation showed an improved anticancer effect in vitro in HepG2 cells via inhibition of their proliferation and promoting apoptosis. The in vivo studies in the HepG2 xenograft model in mice revealed that the developed nanoparticles improved tumor growth suppression, promoted tumor apoptosis and decreased the expression of vascular endothelial growth factor. Gold nanoparticles containing resveratrol were prepared by Chen et al. for the prophylaxis of cataract [115]. The systems possessed a size below 100 nm and negative zeta potential. The nanoformulation showed an improved antioxidant effect in vitro in lens epithelial HLECB3 cells and a more enhanced delay of cataracts in vivo in rat cataract models. In another study, gold nanoparticle–resveratrol conjugates were prepared and possessed approximately 40 nm mean diameter, positive zeta potential and PDI of 0.22 [116]. The anticancer effect of the polyphenol was improved via enhancing the caspase-mediated apoptosis in PANC-1 human cancer pancreatic cells. Resveratrol was loaded into silver nanoparticles coated with albumin reaching approximately 63% encapsulation efficiency [117]. The loading resulted in improved cell uptake and enhanced apoptosis in the breast cancer MCF-7 cell line. Resveratrol was encapsulated into chitosan-modified hollow manganese dioxide nanoparticles via the adsorption method for the treatment of spinal cord injury [118]. The particles possessed 130 nm average size and around 21% drug loading. The loading resulted in improved antioxidant, anti-inflammatory and anti-apoptotic effects in vitro in neuronal PC12 cells and microglial BV2 cells in comparison with the free drug. It was also reported that Fe_3_O_4_/rGO (reduced graphene oxide) magnetic nanocomposites were loaded with resveratrol, which resulted in increased conjugation with calf-thymus DNA compared to the free polyphenol [119]. This could be considered beneficious since it could provide targeting and improve the anticancer, antibiotic and antiviral effects of the drug.

Along with the advantages of the inorganic and metal nanoparticles, there are some limitations that could hinder their application. A recent study reported that mesoporous silica nanoparticles could provoke haemolysis since their silanol groups interact with phospholipids within blood cells [120]. The metal nanoparticles could provoke toxicity, carcinogenicity and irritation [121].

### 4.2. Formation of Nanocrystals

Another approach to dealing with the problematic properties of resveratrol is obtaining nanocrystals. The nanocrystal strategy is vastly appropriate for poorly soluble drugs. The preparations may also increase bioavailability and adhesiveness to the gastrointestinal mucosa and lead to increased uptake. In addition, the low number of excipients in their formulation (1–2%) ensures a low potential for toxic effects. In fact, nanocrystals are produced in the form of aqueous nanosuspension with the addition of a small amount of surfactants, polymers or their mixture. That is why many nanocrystal-based products are approved by the FDA, particularly Fenofibrate (Tricor1^®^), Verapamil hydrochloride (Verelan PM^®^), Diltiazem (Herbesser^®^), Griseofluvin (Gris-Peg^®^), Aprepitant (Emend^®^) and others. These products are intended for various routes of administration, e.g., ocular, parenteral, pulmonary, oral and dermal [122]. Some hydrophobic natural molecules, such as quercetin, apigenin, baicalein, rutin, lutein, epicatechin, hesperetin and hesperidin, were formulated as nanocrystals using different methods and stabilizers, such as Tween 80, Poloxamer 188, Sodium dodecyl sulphate, etc. [123]. Their bioavailability was improved by decreasing particle size and increasing dissolution rate. Nanocrystals of resveratrol using Pluronic F127 were obtained by the wet media milling technique, and the formulation showed better anticancer activity (inhibition of tumor growth, total number of tumor cells, concentration of NO and arginase activity, increased percentages of active macrophages) and antiangiogenic activity (inhibition of VEGF and metalloproteinases (MMP-2)) than the pure substance. Furthermore, obtaining nanocrystals is an approach to protecting resveratrol in vivo from fast metabolism and excretion [124]. Argenziano et al. used the pearl milling method to produce resveratrol nanocrystals that were further coated with soybean lecithin [125]. The authors observed increased dissolution, prolonged retention time of resveratrol in rat plasma and a 3-fold increase in the AUC, leading to improved bioavailability. Another study group also prepared nanocrystals from resveratrol by the sonication method using D-α-Tocopherol polyethylene glycol 1000 succinate as a stabilizer and Pluronic F127 as a co-stabilizer [126]. The authors reported an increased cytotoxic effect on breast cancer cells in vitro and improved oral bioavailability (3.5-fold larger AUC and 2.2-fold higher Cmax) of the nanocrystals. Nanocrystals of resveratrol using hydroxypropyl methylcellulose as a stabilizer were obtained via the antisolvent precipitation method [127]. The neuroprotective effect of the polyphenol was enhanced by increased cellular uptake and permeability of the nanocrystals in contrast with the pure substance. In vivo experiments also confirmed higher plasma and brain concentrations of the nanocrystal form. Another study group prepared nanocrystals by applying the anti-solvent method and using Poloxamer 188, polyvinylpyrrolidone-K30, hydroxypropyl methylcellulose and sodium dodecyl sulfate as stabilizers [128]. They were further formulated into microneedles for transdermal delivery. The solubility of resveratrol was increased 14.61 times, and improved skin permeability was achieved. Despite the advantages of this approach, the main drawback of nanocrystals is related to instability problems that could occur during the preparation techniques such as particle aggregation, amorphization or crystallization [129].

### 4.3. Complexation

Preparation of complexes is a well-known approach for the improvement of the issues of hydrophobic drugs. For instance, complexes of resveratrol and proteins may improve the aqueous solubility, stability and bioavailability of the drug [130]. The study reported the complexation of resveratrol and soy protein isolate that improves the solubility of the polyphenol and its antioxidant activity. Other complexes between resveratrol and proteins were also prepared, and the stability of the drug has been improved, particularly with collagen [131], with β-lactoglobulin, which improved the solubility of resveratrol and slightly its photostability [132] and with caseinate or caseinate–dextran, which protected the drug from isomerization [133].

Other types of complexes are those between hydrophobic drugs and cyclodextrins or their derivatives. These compounds have a specific ring-shaped structure consisting of bonded α-D-glucopyranoside units that form the hydrophilic outer surface of the ring and hydrophobic cavity. The ability of cyclodextrins to increase the aqueous solubility and stability of hydrophobic drugs is mainly due to the inclusion of the drugs in the cavity. There are various products of cyclodextrin complexes in pharmaceutical practice, particularly Prostavasin (intravenous solution based on α-cyclodextrin complex), Nicorette (sublingual tablet containing β-cyclodextrin complex), Voltaren ophtha (eye solution based on 2-hydroxypropyl-γ-cyclodextrin) and others [134]. Complexes of resveratrol and cyclodextrins have also been prepared, achieving higher aqueous solubility, enhanced anticancer activity, wound healing ability and stability [62,135,136,137,138,139]. Another advantage of cyclodextrin complexes is that they can be used as enhancers of encapsulating hydrophobic drugs like resveratrol in various nanoparticles [58,140,141,142]. For instance, a cyclodextrin–resveratrol inclusion complex was incorporated into hydrophilic compartments of liposomes, which led to the complete release of the drug for 24 h [140]. In another study, resveratrol was formulated in a hydroxypropyl-β-cyclodextrin complex that was further incorporated into chitosan-albumin nanogel [58]. The nanocomposite system enhanced the cardioprotective and neuroprotective effects of the polyphenol. Interestingly, Jeong et al. developed an inclusion complex between resveratrol and cycloamylose [143]. It had the same primary structure as cyclodextrins but higher aqueous solubility and larger hydrophobic cavity, which resulted in a 6000-fold increase in the solubility.

Therefore, the complexation of resveratrol is a promising strategy for increasing its aqueous solubility, which results in enhanced pharmacological effects as well as in facilitated incorporation into hydrophilic nanoparticles. The possible drawbacks of this approach are related to the sterically hindered formation of the complex as well as involvement of the active groups of the drugs in the complex, which could lead to compromised effects.

### 4.4. Derivatives and Analogues of Resveratrol

**Figure 5:**
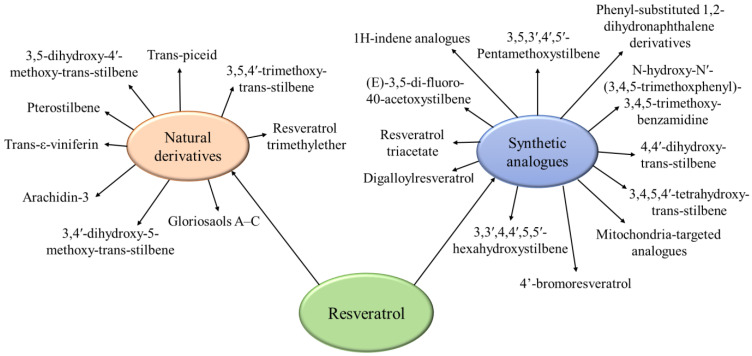
Examples of natural derivatives and synthetic analogues of resveratrol.

The substitution of resveratrol with its derivatives or analogues could be considered as a strategy for the improvement of its limitations. The natural derivatives of resveratrol have a similar structure and are present in food and plants, while some analogues are synthesized (Figure 5).

#### 4.4.1. Derivatives

Methoxylated derivatives are widely presented natural substances. Oral administration of resveratrol and its methoxylated derivative pterostilbene (3,5-dimethoxy-4′-hydroxy-trans-stilbene) in rats showed that the plasma levels of pterostilbene and pterostilbene sulfate were greater in comparison with resveratrol and its sulfated metabolite [144]. The bioavailability of the methoxylated derivative was 80% vs. 20% for resveratrol. Furthermore, it was confirmed that pterostilbene is more efficient as a modulator of cognition and cellular stress in aging and Alzheimer’s disease in lower doses than resveratrol [145]. Interestingly, the intracellular levels of pterostilbene in three types of colon cancer cell were 2–4-fold higher than those of resveratrol, and the derivative had a more intensive inhibitory effect on the cells [146]. Another methylated derivative of resveratrol—3,5,4′-trimethoxy-trans-stilbene—showed improved pharmacokinetic characteristics, particularly lower clearance, longer elimination half-life, higher exposure in plasma and a very well expressed anti-inflammatory effect in vitro [147,148]. Peñalver et al. also confirmed that methylated and butylated resveratrol derivatives have better neuroprotective and anti-inflammatory effects in vitro than resveratrol [149].

#### 4.4.2. Analogues

Modification of the molecule may lead to improved activities. In particular, 3,4-dihydroxyl groups can enhance the antioxidant and anti-tumor activities of resveratrol [17]; dimerization, halogenation and the presence of the hydroxyl group in conjunction with the methoxy group showed better antibacterial activity [35]; and hydroxylated analogues have increased selective COX-2 inhibition activity [150]. For example, oxyresveratrol (a hydroxylated analog of resveratrol) showed higher inhibiting activity in human bladder cancer T24 cells, which indicated that the increased number of phenolic hydroxyl groups augmented the anticancer activity [151]. However, the authors found that the anticancer activity could also be enhanced by the metabolites of the analogues. They observed that acetylresveratrol possessed stronger antitumor activity than resveratrol, although the three phenolic hydroxyl groups are replaced by acetyls. The explanation is that the mean metabolite of this analogue is exactly resveratrol. In particular, in in vivo media the acetyl groups of the applied acetylresveratrol initially protected the phenolic hydroxyl groups from sulfation or glucuronidation, and after that the hydrolysis of acetyls resulted in resveratrol with available phenolic groups [151]. Moreover, analogues of resveratrol possessing improved stability and better aqueous solubility were synthesized, and their anti-inflammatory effect was tested [152]. All the analogues showed such an effect, but pyridyl-substituted ones and the Mannich bases had the highest potential. Synthetic analogues of resveratrol containing 1,3,4-oxadiazole and amide moieties were found to show superior antibacterial activities against *Xanthomonas oryzae* pv *oryzicola* and *Xanthomonas oryzae* pv *oryzae* [153]. Namely, one of these analogues demonstrated significantly more effective inhibitory concentration against both species (4.2 and 5.0 mg/L) in comparison with 63.7 and 75.4 mg/L for free resveratrol.

In conclusion, some derivatives and analogues of resveratrol possess better pharmacokinetics and pharmacodynamics properties, aqueous solubility, stability, bioavailability and finally improved activities. This makes them promising agents for overcoming resveratrol’s limitations and enhancing the effectiveness of therapies. The challenge related to the derivatives and analogues is that the changes in the chemical structure could lead to different pharmacological as well as toxicological profiles. Usually, comparison between the biological properties of the new analogues and the known compound are needed [154].

### 4.5. Prodrugs

Prodrugs are derivatives of active substances that undergo in vivo transformation (chemical or enzymatic) and become the drug itself. The aim of these compounds is to improve the biopharmaceutical and physicochemical properties of drugs and to enhance the pharmacological effect. Due to this strategy, about 7% of the approved drugs are in a form of prodrugs. Hydroxyl, amine, carboxylic, carbonyl and phosphate/phosphonate groups are the most common targeted functional groups that can be modified in order to obtain prodrugs. Using this approach, lipophilicity or permeability (e.g., monoethyl ester of enalaprilat, ethyl ester of oseltamivir carboxylate) and aqueous solubility (e.g., phosphate ester of prednisolone) can be improved [155]. To fulfil these improvements, the prodrug of resveratrol should be metabolized to high plasma concentrations and enter specific tissues, which can metabolize it to provide high tissue concentrations, enhance permeation, prevent first-pass metabolism and conjugative modifications during absorption [156,157]. For instance, in order to inhibit the fast metabolism via conjugation (phase II) and to improve the physicochemical properties of resveratrol, Mattarei et al. synthesized a prodrug in which the hydroxyl groups are engaged with a natural amino acid, isoleucine, in an N-monosubstituted carbamate ester [158]. The prodrug showed good solubility and stability, protected resveratrol from first-past metabolism and provided sustained delivery. The disadvantage of the formulation was the low absorption after oral administration in rats, probably due to the high hydrophilicity of the three carboxylic ionisable groups [158]. Other prodrugs of resveratrol with the OH group engaged in formal (–OCH2OR) or acetal (–OCH(CH3)OR) linkages, where R is ethyleneglycol oligomers (OEG) with terminal methoxy group –O–(CH2CH2O)n–CH3 (n = 0, 1, 2, 3, 4, 6), were also synthesized [159]. The prodrug with the formal bonds was too stable and could not regenerate the parent drug at a high enough rate. The prodrug with the acetal bond protected resveratrol from phase II metabolism and provided sustained absorption [159]. An intragastric administration of the prodrug of resveratrol (3,5,4′-Tri-O-acetylresveratrol) showed prolonged t_1/2_ and increased AUC [160]. It was distributed in spleen, heart, liver and lung, where the concentrations of resveratrol were significantly increased. An alkylated prodrug of resveratrol, particularly 3-O-(6′-O-octanoyl)-β-D-glucopyranoside resveratrol, was also obtained in order to increase its efficacy (through structural modulation) and to improve its bioavailability (through the prodrug prepared) [149]. The prodrug showed an enhanced neuroprotective effect on zebrafish, improved onset and symptoms of Huntington’s disease in a mice model and better anti-inflammatory and antioxidant action than resveratrol. The authors claim that the octanoic chain in the structure of the prodrug may also have an influence on these effects. The prodrug 3,5-triethylsilyl-4′-(6″-octanoylglucopyranosyl) resveratrol was also found to be more active in a Huntington’s disease mice model and a mice model of multiple sclerosis [161].

Finally, synthesizing prodrugs of resveratrol is a promising approach, but it has to be further developed considering some drawbacks related to physicochemical and biopharmaceutical issues. The time-consuming synthesis of prodrugs could also be considered a challenge [162].

## 5. Conclusions

Thus, it can be concluded that resveratrol shows numerous positive effects and therefore it could be used as a promising therapeutic in the treatment of different health conditions. On the other hand, the low aqueous solubility and stability of resveratrol significantly affects its biopharmaceutical profile and finally compromises its effects. For this reason, new promising approaches to cope with these obstacles, which may be considered the future of modern medicine, are being intensively researched. The improved solubility of resveratrol could lead to more effective intracellular transport and a lower dose of administration. The latter is very important, having in consideration the reported adverse effects of resveratrol, e.g., the cytotoxic concentration for various cancer cells is also toxic for normal cells. Moreover, the proceeding innovative formulations of resveratrol in clinical trials for the treatment of different conditions could be considered as a future perspective. Thus, it is important to evaluate the potential of each of the developed strategies and overcome their limitations.

## Figures and Tables

**Table 3:** Types of inorganic nanoparticles as carriers of resveratrol and their advantages.

Type	Advantages	Reference
Mesoporous silica nanoparticles	Increased solubility Enhanced anticancer effect	[111]
Lactoferrin functionalized mesoporous manganese doped silica nanoparticles	Enhanced antioxidant and anti-inflammatory effect Improved bioavailability	[112]
Mesoporous silica nanoparticles	Enhanced anticancer effect	[113]
Metal (gold) nanoparticles	Improved antitumor effects	[114]
Metal (gold) nanoparticles	Enhanced antioxidant effects	[115]
Metal (gold) nanoparticles	Improved antitumor effects	[116]
Metal (silver) nanoparticles	Improved antitumor effects	[117]
Metal (manganese dioxide) nanoparticles	Improved antioxidant and anti-inflammatory effects	[118]
Magnetic (Fe_3_O_4_/rGO) nanoparticles	Improved interactions with thymus DNA	[119]

## Data Availability

All data are contained within the article.
